# Adaptation of Fe-S Cluster Assembly to Rising O_2_ Levels over Geological Time

**DOI:** 10.21203/rs.3.rs-7916008/v1

**Published:** 2026-01-08

**Authors:** Hailiang Dong, Hongyu Chen, Franklin Outten, Li Huang, Zhenfeng Zhang, Daijiang Xiong, Mengtong Zhang, Xiaoqin Tang

**Affiliations:** China University of Geosciences; China University of Geosciences (Beijing); University of South Carolina; Southern Marine Science and Engineering Guangdong Laboratory; Institute of Microbiology, Chinese Academy of Sciences; State Key Laboratory of Microbial Resources, Institute of Microbiology, Chinese Academy of Sciences; China University of Geosciences; China University of Geosciences

## Abstract

One of the most important events in Earth history is the Great Oxidation Event (GOE). While O_2_ killed most anaerobic microorganisms, some survived. Fe-S clusters are cofactors essential for cellular processes in all life forms, but how they adapt to rising O_2_ remains unclear. Sulfur utilization factor (SUF) pathway is one of the most common Fe-S assembly pathways. We hypothesize that within the SUF pathway, SufE, as a sulfur-transfer partner of cysteine desulfurase SufS, maintains its functions under oxidative stress through molecular adaptation. Molecular clock dating showed SufE originated ~2.67 Ga (i.e., last common ancestor, LCA) and diversified considerably around the GOE (~2.14 Ga). The corresponding ancestral SufS was also reconstructed for these two times. Biochemical assays reveal that SufS^LCA^/SufE^LCA^ is active at up to ~2% O_2_, higher than Archaean atmospheric O_2_, whereas SufS^GOE^/SufE^GOE^ is active at up to ~10% O_2_, higher than the level during the GOE. These advanced evolutions may have provided resilience to redox fluctuations through Earth history. Growth experiments showed that overproduction of either SufE^GOE^ or SufS^GOE^/SufE^GOE^ in *Escherichia coli* mutants lacking SufE or SufSE better restores its growth than overproduction of their LCA counterparts, consistent with the in vitro results. Enzyme structure prediction revealed that such adaptation was achieved through replacement of a few amino acids in key catalytic sites and consequent conformational changes of key enzymes. Our results reveal the molecular mechanism of adaptation of Fe-S cluster assembly to rising O_2_ and significantly contributes to the coevolution of the geosphere and biosphere.

One of the most important events in Earth history is the so-called Great Oxidation Event (GOE)^1,2^. It refers to a time period (between 2.46–2.06 Ga) when the Earth atmosphere and shallow sea experienced a rise in the free O_2_. During the latter half of GOE (i.e., Lomagundi-Jatuli Event, LJE, 2.22–2.06 Ga), atmospheric O_2_ levels experienced a marked but temporary increase^3,4^. While accumulation of free O_2_ killed the majority of anaerobic microorganisms, sometimes termed the first mass extinction event^5^, some survived through evolution to either tolerate or utilize O_2_^6^. However, the molecular mechanisms underlying the evolution remain to be understood. Analysis of a biological process prevalent in extant life and yet ancient and sensitive to molecular oxygen may offer valuable clues.

Iron-sulfur (Fe-S) clusters are such ancient cofactors. They are essential for a wide range of enzymes and metabolic processes, such as DNA repair and electron transfer^7–9^. Biosynthesis of Fe-S clusters requires multiprotein assembly systems that mobilize sulfur, iron, and electrons to construct clusters and insert them into target apoproteins^9^. In *Escherichia coli*, two major Fe-S assembly pathways exist: the housekeeping ISC system, functioning primarily under normal conditions, and the stress-responsive SUF system, which is induced under oxidative stress and iron limitation^10–13^.

Comparative phylogenomic analysis indicates that the SUF pathway is one of the oldest Fe-S biogenesis machineries: minimal SUF-like systems are implicated in the last universal common ancestor and distributed among Bacteria and Archaea^14^. This broad distribution underscores the functional plasticity of SUF and suggests that this pathway played a central role in early microbial adaptation to fluctuating redox conditions. Notably, stress-inducible SUF systems (such as that in *E. coli*) utilize SufE as the sulfur-transfer protein. Rate-smoothed phylogenies further indicate that the emergence of accessory cysteine desulfurase SufS and its sulfur-carrier partner SufE were triggered by oxygenation of the Earth^15^. However, the timing of their emergence remains uncertain and warrants further study.

SufE is a small sulfur-transfer protein of the SUF system. In *E. coli*, SufE accepts a sulfur atom from the cysteine desulfurase SufS and then delivers it to the scaffold complex (SufBC_2_D) for Fe-S cluster assembly^16,17^. This transient SufS/SufE interaction is vital for efficient sulfur transfer^18^. SufE forms a hydrophobic pocket to protect cysteine-persulfide from oxygen in aqueous solution^19^. Indeed, SufS and SufE have been shown to be crucial for bacterial survival under oxidative stress conditions in *E. coli*^20^. However, how SufS/SufE has evolved in response to rising atmospheric O_2_ over geological time remains unknown. We hypothesize that oxidative pressure favored genetic mutations in SufE and SufS to maintain Fe-S biogenesis in an increasingly oxidizing world. We focused on SufE because of its central role in sulfur transfer under oxidative stress and its small size, which would allow relatively rapid evolutionary adaptation^14^. We also examined SufS, as the SufS/SufE cooperating pair likely underpins the functionality of the Suf pathway under oxidative stress^20–22^.

In this study, we investigated the O_2_-driven evolution of SufS/SufE. We performed phylogenetic analyses and molecular clock dating to predict the emergence and diversification of SufE in the context of the oxygenation history of Earth, and reconstructed ancestral sequences of SufE (and SufS) corresponding to two key time points, i.e., ~2.67 Ga, when SufE originated, and ~ 2.14 Ga (at the LJE). Our data reveal how O_2_ has shaped the evolution of the proteins, and therefore provide significant insights into molecular events in the explosion of aerobic life through Earth history.

## SufE emergence before the GOE

**Figure 1:**
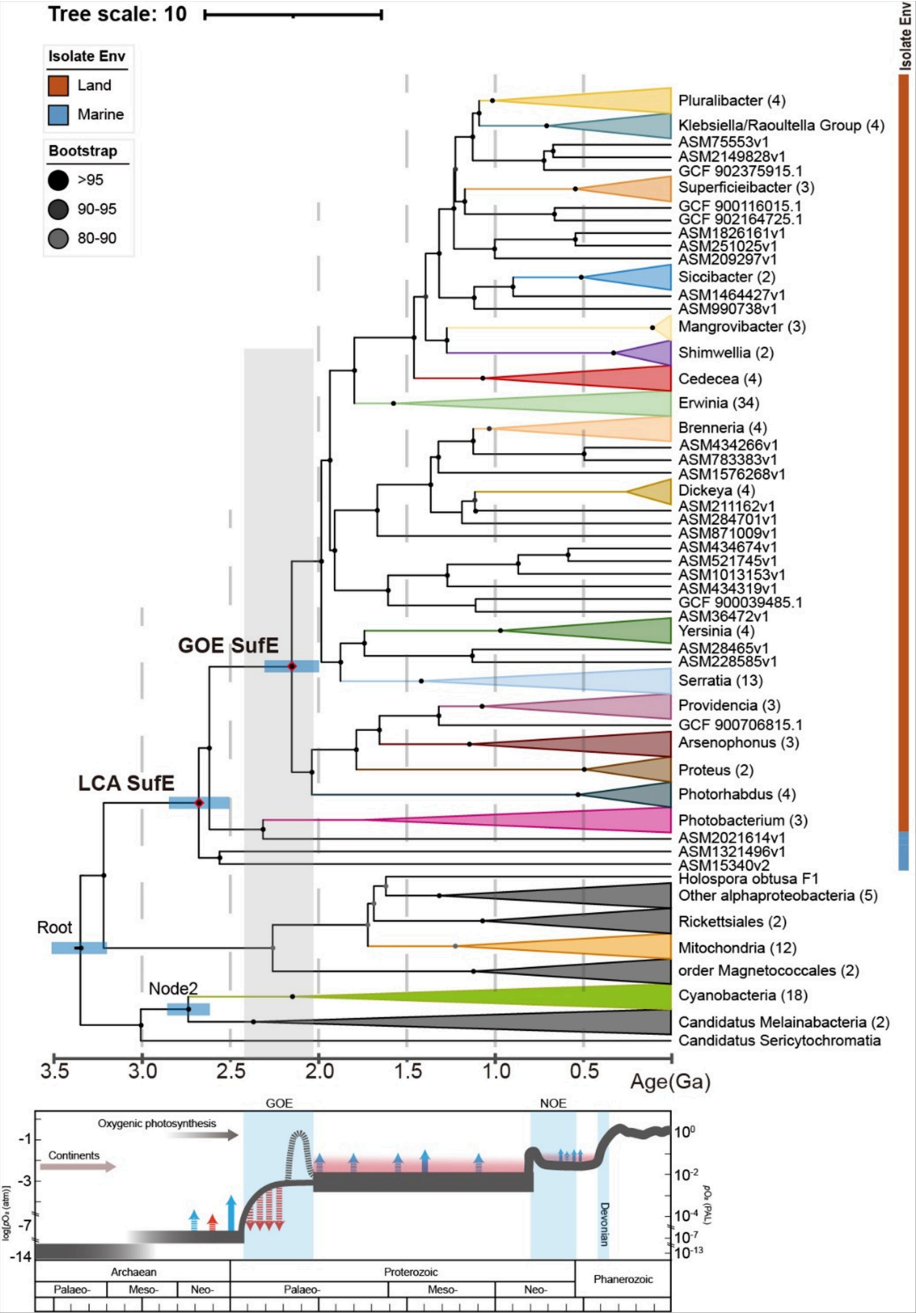
Phylogenetic analysis of SufE evolution in the context of Earth’s oxygenation. A time-calibrated phylogenetic tree of SufE proteins from diverse bacteria is shown with a geological timeline. Diamonds at key nodes indicate reconstructed ancestral sequences. The timeline below the tree illustrates atmospheric O_2_ levels over Earth’s history (blue line), highlighting the Great Oxygenation Event (GOE, ~2.4–2.0 Ga) and the Neoproterozoic Oxygenation Event (NOE, ~0.6–0.8 Ga) with shaded regions^1^. The deepest branches of the SufE tree (“Last Common Ancestor (LCA) SufE”, the corresponding protein named SufE^LCA^ hereafter) predate the GOE, suggesting SufE existed in anoxic environments. A major diversification of SufE (“GOE SufE”, the corresponding protein named SufE^GOE^ hereafter) aligns with the GOE (grey shading). Calibration lineages are noted (*e.g*., mitochondria-derived sequences in orange, and cyanobacteria in green). Others are annotated with their habitat (*e.g*., marine or terrestrial). The dating genome information was stored at github.com/eacochen. Bootstrap support values for key branches are indicated by black circles, and all values are higher than 95%. The analysis suggests that O_2_ availability influenced the evolutionary radiation of SufE.

We constructed a maximum-likelihood phylogeny of SufE homologues from over 7,000 prokaryotic genomes (Extended Data Fig. 1). Among these basal candidate lineages, we chose Gammaproteobacteria clade II for molecular dating because (i) phylogenetic reconciliation of 120 single-copy core genes with *sufE* yielded fully congruent topologies, indicating strictly vertical inheritance with only one detectable horizontal gene transfer (HGT) event and (ii) the clade spans a well-resolved evolutionary gradient in heme-copper oxidase (HCO) types responsible for O_2_ reduction to water. The basal lineages encode microaerobic C-type HCOs, whereas the more recently derived lineages possess fully aerobic A-type HCOs. This stepwise shift from microaerobic to aerobic respiration provides an ecological framework that directly links divergence times to progressive adaptation to increasing oxidative stress (Extended Data Figs. 1–2). Bayesian relaxed molecular clock analyses using the autocorrelation rates (AR) model placed the last common ancestor (LCA) of SufE (Fig. 1, hereafter named SufE^LCA^), represented by the basal lineage of Gammaproteobacteria clade II near the root of the SufE phylogeny, at 2.67 Ga (95% Highest Posterior Density, HPD: 2.49–2.83 Ga), shortly after the inferred origin of oxygenic cyanobacteria (~ 2.73 Ga) (Figs. 1, Extended Data Fig. 3–4). This temporal proximity implies that SufE evolved in response to the “whiffs of O_2_”, potentially preceding the global-scale GOE. This result suggests that the SufS/SufE system is an ancient invention, emerging between the appearance of oxygenic cyanobacteria and the advent of O_2_^14^.

Subsequent branching patterns indicate that major diversification or expansion of the SufE family occurred at the latter half of the GOE, *e.g*. LJE (Fig. 1, hereafter named SufE^GOE^). Notably, the SufE^GOE^ node represents the earliest SufE-carrying lineage isolated from terrestrial environments and was dated to 2.14 Ga (95% HPD: 1.98–2.29 Ga). This age coincides with the time of thickening of the continental crust^23^ and rising O_2_ levels, suggesting that SufE proteins were under selective pressure to accommodate elevated oxidative stress during the early colonization of land niches. We interpret this as evidence that the selective pressure from a rise in O_2_ led to rapid diversification of SufE sequences and/or a selective sweep of O_2_-tolerant SufE variants around that period.

Together, these results suggest that SufE underwent rapid evolutionary divergence under early oxidative stress, likely adapting to the sporadic O_2_ “whiffs” produced by nascent cyanobacteria before the GOE^1^ and to the rapid increase of O_2_ during the LJE period. We set out to test if the changes in SufE sequence are bona fide molecular adaptations that resulted in improved SufE function under oxidative stress. Our approach involves ancestral sequence reconstruction coupled with the comparison of ancestral and existent proteins in biochemical and functional assays.

## In vitro response of SufSE activity to O_2_

Two nodes in the SufE phylogenic tree, LCA and GOE SufE, were selected for detailed ancestral sequence reconstruction because they bracket the GOE, permitting a direct test of a functional shift in response to an increase in O_2_ level. Three SufS/SufE pairs, corresponding to SufS^LCA^/SufE^LCA^, SufS^GOE^/SufE^GOE^ and SufS^modern^/SufE^modern^ (*E. coli* K12), were expressed in *E. coli* and purified under anerobic conditions. The reconstructed and modern sequences were aligned (Extended Data Figs. 5–6). The core catalytic residues, such as C51 in SufE^modern^ (corresponding to both C51 in SufE^GOE^ and SufE^LCA^, Extended Data Fig. 5) and C364 in SufS^modern^ (corresponding to C369 in SufS^GOE^ and C368 in SufS^LCA^, Extended Data Fig. 6), have remained unchanged over time. However, there are mutations in the loop between β1 and β2 of SufE, sites of interaction between SufS and SufE, and the interface of the SufS homodimer (Extended Data Figs. 5–6).

**Figure 2:**
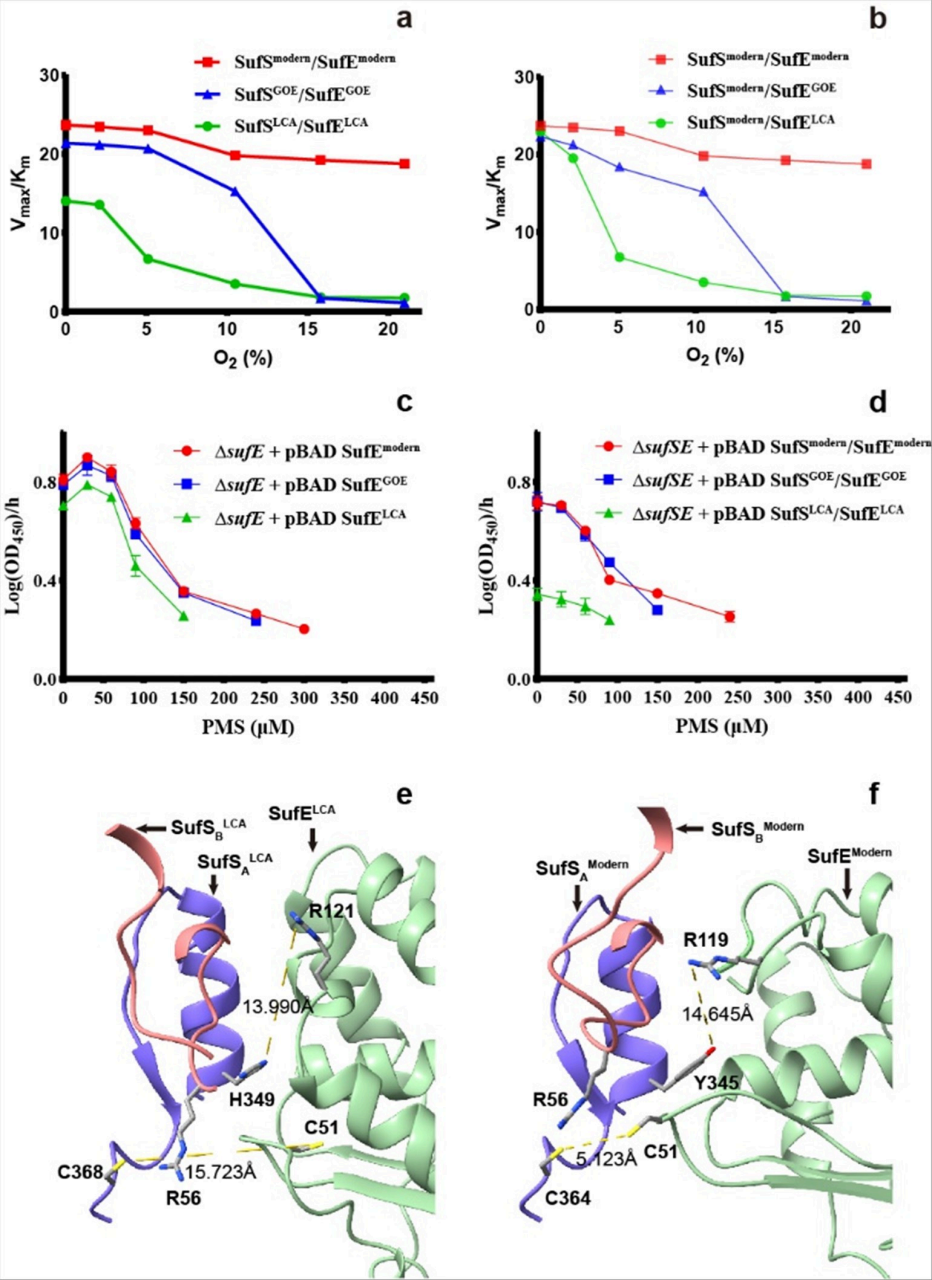
O_2_ tolerance of ancestral and modern SufS/SufE complexes. **(a-b)**: Catalytic efficiency (Vmax/Km) of the LCA (green), GOE (blue), and modern (red) SufS/SufE proteins as a function of O_2_ concentration. Each point represents the mean (± s.d.) of three experiments and error bars are too small to be visible. **(a)**: Matched SufS/SufE pairs. **(b)**: *E. coli* K12 SufS^modern^ paired with either SufE^LCA^, SufE^GOE^ or SufE^modern^ variants. The K_m_ of SufE was determined as follows: SufS concentration was fixed at 0.5 μM and L-cysteine at 500 μM, while SufE was varied over a range (*e.g*. 0–50 μM). According to the method, the K values of SufE for each SufS/SufE pair are as follows: modern pair showed K ≈3.64 μM; GOE pair K ≈4.88 μM; LCA pair K ≈2.48 μM. **(c-d)**: The maximum growth rate (per hour) of *E. coli* strains lacking either SufE (WO541, *E. coli* MG1655 Δ*iscU-fdx* Δ*sufE::cm*^R^ *MVA*^*+*^*-kan*^R^ mutant) or both SufS and SufE (WO539, *E. coli* MG1655 Δ*iscU-fdx* Δ*sufSE::cm*^R^ *MVA*^*+*^*-kan*^R^ mutant), but complemented with ancestral or modern variants carried by pBAD, in the presence of the superoxide-generating agent phenazine methosulfate (PMS) (0–450μM). **(c)** Δ*sufE* strain WO541 complemented with SufE^modern^ (red), SufE^GOE^ (blue), or SufE^LCA^ (green). **(d)** Δ*sufSE* strain WO539 complemented with SufS^modern^/SufE^modern^ (red), SufS^GOE^/SufE^GOE^ (blue), or SufS^LCA^/SufE^LCA^ (green). Data points are mean ± s.d. for biological replicates (n = 3). **(e-f)**: Hypothetical structural model for SufS/SufE. A schematic model illustrating the interaction between the SufS homodimer (red and blue) and SufE (green). **(e)** AlphaFold3 predicted structure. H349 is present in SufS^LCA^, which prevents the catalytic two cysteine residues between SufS and SufE from approaching each other (distance 15.723 Å), this accounting for its low catalytical efficiency of persulfide transfer. **(f)** “Close approach” phase shows how SufS^modern^ interacts with SufE^modern^ adapted from Gogar, et al.^18^. In this case, the presence of Y345 on SufS allows a close approach between the catalytic two cysteine residues to allow efficient transfer of persulfide (distance 5.123 Å).

To determine the function of LCA, GOE and modern SufS/SufE pairs in response to rising O_2_, each pair was assayed for cysteine desulfurase activity by detecting sulfide production across an O_2_ gradient (0–21%). All three variants of SufE greatly enhanced the low basal activity of SufS (data not shown), presumably by acting as a persulfide acceptor, thereby facilitating SufS turnover, consistent with previous studies for the modern pair^20^. Furthermore, under strictly anoxic conditions (0% O_2_), all three SufS/SufE pairs were active, but the modern pair exhibited the highest catalytic efficiency (Fig. 2a). Both the modern *E. coli* and the GOE pairs showed a V_max_/K_m_ ratio ~ 40–50% greater than that of the LCA pair.

However, the three pairs exhibited markedly different patterns in response to O_2_. The activity of the LCA pair decreased precipitously at 2% O_2_ (Fig. 2a, green curve), fell to less than half of its anoxic value at 5% O_2_, and essentially dropped to the baseline level of SufS at 10% O_2_. The SufS^GOE^/SufE^GOE^ (Fig. 2a, blue curve) largely retained its anoxic activity at low O_2_ levels (i.e., ~ 90% activity at 5% O_2_ and ~ 70% at 10% O_2_) but dropped to the baseline level of SufS at 15% O_2_. In stark contrast, the modern pair from *E. coli* K12 (Fig. 2a, red curve) retained its full activity up to ~ 5% O_2_ and only dropped to ~ 80% at the modern O_2_ level (21%). These results indicate a stepwise improvement in O_2_ tolerance from the LCA to the GOE to the modern variants.

We then determined if SufS or SufE was primarily responsible for the O_2_ sensitivity by pairing the SufS^modern^ with either the SufE^LCA^ or the SufE^GOE^ (Fig. 2b). Under anoxic condition, the SufS^modern^/SufE^LCA^ combination increased the activity by ~ 100% relative to the SufS^LCA^/SufE^LCA^ combination, yielding a catalytic efficiency indistinguishable from the SufS^modern^/SufE^modern^ pair. Therefore, it is SufS that determines the maximal catalytic efficiency of the full SufS/SufE transpersulfuration reaction. As O_2_ concentration increased, the activities of the three hybrid combinations show similar response patterns (Fig. 2b) to those of the three “age-match” combinations (Fig. 2a). It appears that, when SufS was kept unchanged (modern variant), different SufEs caused the difference in O_2_ sensitivity among the three hybrid SufS/SufE pairs (Fig. 2b).

To understand the structural basis of the difference in catalytic activity among the three SufS/SufE pairs, AlphaFold3 was used to predict the structures of SufS and SufE complexes based on a rigid docking model^18^. In the SufS^LCA^, a small and flexible histidine is present at position 349 (corresponding to 350 in SufS^GOE^ and 345 in SufS^modern^, Extended Data Fig. 6)^24^. This residue, H349, fails to occupy the R121 cavity of SufE^LCA^ (corresponding to R121 in SufE^GOE^ and R119 in SufE^LCA^) and allows R121 to stay at a blocking position (Fig. 2e). As a result, the distance between C368 of SufS^LCA^ and C51 of SufE^LCA^ (i.e., 15.723 Å) is sufficiently large to slow down the persulfide transfer from SufS to SufE, thus accounting for its low catalytical activity. In contrast, in SufS^modern^ (also in SufS^GOE^), a larger and more rigid tyrosine replaces histidine at position 345 (Extended Data Fig. 6) and occupies a cavity vacated by the C51 loop of SufE^modern^. Consequently, R119 in SufE^modern^ is forced to move toward an outward position, allowing C51 of SufE^modern^ to approach C364 of SufS^modern^ for rapid persulfide transfer (Fig. 2f)^18^. Thus, the increased catalytical activity of the modern and GOE pairs, relative to the LCA pair (Fig. 2a), is likely triggered by the replacement of histidine in SufS^LCA^ by tyrosine in SufS^GOE^ and SufS^modern^ to result in a more efficient interaction between the SufS and SufE catalytic sites. There are other residue-level substitutions along the β-latch/homodimer interface of SufS (i.e., K92 in SufS^GOE^ versus R92 in SufS^LCA^ and SufS^modern^ variants, Q255 in SufS^LCA^ versus E255 in SufS^GOE^ and E250 in SufS^modern^ variants), but they do not appear to compromise the monomer-monomer interaction of SufS (Extended Data Fig. 7) and the ability of SufS to interact with SufE.

In addition to these substitutions on SufS, there are numerous differences among the three variants of SufE (Extended Data Fig. 5) that may jointly account for the measured differences in their catalytic activities among the three SufS/SufE pairs (Fig. 2a–b). For example, relative to the SufE^modern^, the SufE^LCA^ and SufE^GOE^ show insertions of two amino acid residues between β1 and β2, as well as numerous substitutions both between and within these β-strands (Extended Data Fig. 5). Although these sites are spatially distant from the SufS/SufE interface, they may perturb the overall conformation of SufE, thereby modulating its association with SufS and, in turn, the capacity of SufS/SufE pairs to withstand oxidative stress (Fig. 2).

## In vivo response of SufSE activity to O_2_

To determine the response of SufS and SufE activity to rising O_2_ levels in vivo, we analyzed the functionality of the SufS^LCA^/SufE^LCA^ and SufS^GOE^/SufE^GOE^ pairs in *E. coli* K12. First, we tested if these variants could rescue the synthetic lethality of strains lacking both the housekeeping Isc system and either SufE or both SufS/SufE. Normally, such mutants are inviable due to loss of Fe-S cluster-dependent isoprenoid biosynthesis^25^. Conditionally lethal Δ*iscU-fdx* Δ*sufE::cm*^R^ and Δ*iscU-fdx* Δ*sufSE::cm*^R^ mutant strains were constructed by inserting a non-native, hybrid mevalonate-dependent MVA system that does not require any Fe-S cluster enzymes for isoprenoid biosynthesis^26^. These strains absolutely rely on the non-native MVA system and addition of mevalonate to the growth media in order to restore isoprenoid biosynthesis. We then tested if introducing either ancient SufE alone (SufE^LCA^ or SufE^GOE^) or ancient SufS/SufE pair (SufS^LCA^/SufE^LCA^ or SufS^GOE^/SufE^GOE^) into a pBAD plasmid could allow the conditionally lethal strains to grow without mevalonate. We found that all SufE variants or SufS/SufE pairs rescued the lethality of the strains in the absence of mevalonate under atmospheric O_2_ conditions (i.e., at the origin, Fig. 2c–d). We surmise that basal intracellular oxidative stress at atmospheric O_2_ concentrations was probably not high enough to distinguish their ability in Fe-S cluster biogenesis, especially in the rich, glucose-supplemented media used for the experiment.

To increase the oxidative stress, the Δ*iscU-fdx* Δ*sufE::cm*^R^ strains transformed with the three variants of SufE on the pBAD plasmid were subjected to phenazine methosulfate (PMS). PMS generates intracellular superoxide radicals, imposing extra oxidative stress that damages Fe-S clusters and necessitates active repair/biogenesis systems^27^. Indeed, growth assays revealed clear differences in functional restoration of the three SufE variants under increasing PMS concentration (Fig. 2c). Across 0–30 μM PMS, all complemented strains exhibited a modest stimulatory response, with the maximum specific growth rate (μmax) increasing and peaking at 30 μM. From 30–60 μM, the μmax values declined modestly yet remained above that for the no-PMS control. Above 60 μM, the μmax values dropped sharply for all variants, and the resulting viability limits separated the three lineages: the SufE^LCA^ ceased growth above 150 μM, the SufE^GOE^ above 240 μM, and the *E. coli* K12 SufE^modern^ above 300 μM. Thus, while the low dose stimulation is shared, the SufE^modern^ extends the tolerable PMS window to a much higher level of PMS. These in vivo data mirror the in vitro biochemical assay results, showing that the evolutionary enhancements to SufE’s sequence translate into a tangible survival advantage under oxidative stress.

Similar complementary experiments were performed for the Δ*iscU-fdx* Δ*sufSE::cm*^R^ strain lacking both SufS and SufE. The normalized expression levels of SufS and SufE from LCA, GOE and modern strains were similar without PMS (Extended Data Fig. 8). Baseline growth in the absence of PMS showed that the SufS^GOE^/SufE^GOE^ and SufS^modern^/SufE^modern^ supported nearly identical maximum growth rates, both slightly lower (by ~ 0.1 h^−1^) than in the Δ*iscU-fdx* Δ*sufE::cm*^R^ strain complemented with SufE [~ 0.8 log(OD_450_) h^−1^ vs ~ 0.7, Fig. 2c–d]. In contrast, the SufS^LCA^/SufE^LCA^ pair had a μmax value reduced by ~ 50% (Fig. 2d), matching the magnitude of its diminished catalytic efficiency in vitro (Fig. 2a). Unlike the results in the Δ*sufE* strain, no hormesis was observed in the Δ*sufSE* strain: the μmax value declined slightly over 0–30 μM PMS, then fell steeply from 30 to 240 μM (Fig. 2d). Consistently, the upper tolerance limits of PMS were markedly lower in the complemented Δ*sufSE* strain compared to those in the complemented Δ*sufE* strains, which ceased growth above 100 μM, 150 μM, and 240 μM PMS for LCA, GOE, and modern SufS/SufE pairs, respectively (Fig. 2d). Importantly, under higher PMS stress, the modern SufS/SufE consistently outperformed the GOE and LCA variants.

## Implications for life evolution through Earth oxygenation

Our findings reveal a clear evolutionary trajectory in one of the most important biochemical pathways: SUF Fe-S biosynthesis system, driven by Earth’s rising atmospheric O_2_ concentration. The SUF pathway, though present in early anaerobic life, had to be refined to remain functional as the atmospheric O_2_ levels rose. By reconstructing ancient SufS and SufE proteins, we directly observed that the SufS^LCA^/SufE^LCA^ and SufS^GOE^/SufE^GOE^ complexes were poorly adapted to an O_2_-rich environment, while the modern complex was highly O_2_-tolerant. SufS determines the catalytic ceiling, but SufE governs how much oxidative load that the complex can withstand. Thus, neither component is subordinate: evolution jointly optimized SufS and SufE, likely by tuning their global geometries and coordinating a protected handoff of persulfide to SufBC_2_D scaffold. These results underscore the power of O_2_ as an agent of natural selection.

Certain housekeeping SUF systems (e.g., in *Bacillus subtilis*) employ SufU as a SufE analog, serving as the cysteine desulfurase partner for sulfur transfer in those organisms^28^. A histidine 349 is critical for SufS^LCA^ (corresponding to Y345 in SufS^modern^ and Y350 in SufS^GOE^) when it interacts with SufU which requires a Zn^2+^ cofactor to coordinate interactions during persulfide transfer. Phylogenetically, SufU predates SufE (Extended Data Fig. 9). However, bioavailable Zn in Archaean oceans was extremely low^29^.

As a result, SufE may have evolved as a zinc-independent ‘rescue’ module for the SUF pathway under anoxic condition. However, SufS/SufE pair faces a great challenge of Earth’s progressive oxygenation.

Our molecular dating result indeed suggests that once localized O_2_ appeared, molecular adaptation followed rapidly. The first detectable divergence within the SufE clade was only ~ 60 Myr after the median age of oxygenic cyanobacteria, which is a small age gap in the Archaean context. SufE emerged in lineages that likely shared ecological space with the earliest O_2_ producers. Furthermore, the molecular adaptation was not only quick, but the oxidative tolerance of SufS and SufE actually evolved ahead of contemporaneous O_2_ levels. The earliest-divergent SufE^LCA^ already withstands ~ 2% O_2_, exceeding most estimates for ambient O_2_ at this time (~ 0.1–~0.5%)^30,31^. Likewise, geochemical proxies indicate ~ 4% O_2_ around the LJE^3^, but our in vitro biochemical assay showed that the SufE^GOE^ already tolerates ~ 10% O_2_. These systematic overshoots of O_2_ tolerance suggest selection for excess capacity as a safety margin in stress-response machineries, which possibly provides resilience to redox excursions and facilitates the progressive expansion of increasingly aerobic niches. A similar type of advanced preparedness in response to increasing stress is also shown in antibiotic stress-response^32^.

In addition to oxidative tolerance, the increased catalytic efficiency of SufS/SufE from the LCA to GOE/modern variants may reflect selective pressure to increase rates of Fe-S cluster biogenesis in vivo. During aerobic growth, cells experience a constitutive level of Fe-S cluster damage in sensitive enzymes such as dehydratases, requiring a compensatory increased level of cluster biogenesis to maintain cell function^33^. In contrast, during anaerobic growth, Fe-S cluster turnover is much lower and the demand for cluster biogenesis is lower.

From a broader perspective, the O_2_-driven evolution of SufE and SufS exemplifies how life’s molecular machinery has been molded by planetary change. The GOE forced the innovation of more efficient Fe-S cluster assembly tools, which in turn enabled organisms to exploit O_2_ for metabolism while preserving their ancient biochemical capabilities. Without such adaptations, essential processes, including respiration, DNA repair, and metabolic pathways that depend on Fe-S proteins could have failed in an oxygenated world. One particular example of such adaptation is ancient nitrogenases, Fe-S proteins that are used for N_2_ fixation. Molecular dating suggests that Mo-based nitrogenase originated in the anoxic mid-late Archaean age (3.1–2.7 Ga)^34^, but survived the GOE and retained the same basal structure and functions even in modern aerobic microorganisms^35–37^. Conformational change is one important mechanism against oxidative stress, where the Shethna (FeSII) protein binds to the nitrogenase complex to protect it from oxygen damage^38,39^. While the specifics may vary among different functional groups of organisms, our study reveals an important and possibly widespread defense mechanism against oxidative stress through the conformational change of the SufS/SufE interface.

This work illustrates how geochemistry and biochemistry are intertwined: oxygenic photosynthesis leads to atmospheric oxygenation, and such a planetary-scale change drives molecular innovation, which in turn enables new biological capabilities. Our study therefore connects the evolution of a single protein complex to Earth’s largest evolutionary inflection point, even the Cambrian radiation, by way of the O_2_ that links them. This integrative perspective from atmosphere to enzyme to evolution deepens our understanding of how life’s molecular machinery is molded by our changing planet and invites further investigations using ancestral protein reconstruction to explore other episodes where environmental change and biochemical evolution converged.

## Methods

### Molecular clock and Ancestral Sequence Reconstruction

#### Multiple sequence alignment and phylogenetic analysis

Four experimentally validated SufE/CsdE proteins from UniProt (downloaded 24 Oct 2022) with “UniProtKB reviewed” status (P76194, Q9EXP1, B5BA16, Q1C762, P0AGF2) were used as seeds for homology searches against the NCBI NR protein database (June 2022, 483,768,206 non-redundant sequences) using DIAMOND blastp^40^ with an E-value of < 1e-6 and a length filter of 90–170 amino acid residues, yielding 23,567 SufE-like hits. Candidate sequences were verified by HMMER^41^ hmmscan against Pfam^42^ hidden Markov model (HMM) database, and validated sequences were dereplicated with CD-HIT^43^ (90% identity), grouped into 7,635 (NR) clusters. The longest sequence in each cluster was retained. The sequences were aligned with MAFFT^44^ L-INS-i and trimmed using trimAl^45^ with resoverlap 0.55 and seqoverlap 60. Maximum-likelihood (ML) trees were inferred with FastTree^46^ (for rapid exploration) and IQ-TREE 2.2.1^47^ (for focal subsets). Because of the lack of reliable outgroups for SufE, MAD^48^ and MinVar^49^, outgroup-independent methods were employed to allow identification of ancient lineages and candidate nodes for dating and ancestral sequence reconstruction. Visualization was performed with iTOL^50^, and sequences species annotations were fetched from NCBI identical protein group (ipg) database by Entrez^51^, using the most complete genome for each protein cluster using CheckM^52^ completeness estimates. The putative oxygen-respiration capacity of a species was inferred based on the presence of heme-copper oxidase (HCO) families A, B1, C, as detected with hmmscan (E-value < 1e-50), as described^53,54^.

#### Gene-species tree reconciliation with curation of horizontal gene transfer (HGT) candidates

To satisfy the vertical inheritance assumption for node dating and ancestral sequence reconstruction, SufE gene trees were reconciled with species trees, and those lineages which were likely recipients of HGT^55^ were iteratively removed. For the focal lineage (301 proteins and 1,455 candidate genomes), open reading frames were predicted with Prokka^56^, and species phylogeny was built with GTDB-Tk^57^ bac120 single-copy bacterial markers (hmmscan annotation, E-value < 1e-50). Each marker was aligned with MAFFT, trimmed with trimAl, and concatenated. The species tree was inferred under LG+C20+F+I+G in IQ-TREE. Gene-species reconciliation was performed with GeneRax^58^ under species-tree-aware ML, using SPR search and an unconstrained DTL model (radius 5). Leaf-level HGT acceptors, acceptors at internal nodes transferring to leaves, and transfers between deep internal nodes of comparable depth were successively removed. After six iterations, transfers were reduced from 34 to 1, yielding a curated set of 131 vertically inherited SufE sequences and genomes for dating.

#### Molecular clock analyses

To anchor both deep Proterozoic and Phanerozoic timescales, the following two sets of species were used: (i) 21 oxygenic cyanobacteria and 3 melainabacteria (i.e., the cyanobacterial outgroup), and (ii) mitochondria-proximal alphaproteobacteria and a set of mitochondrial markers (mito24)^59,60^. First, 10 alphaproteobacteria genomes were added close to mitochondria, built a bac120 species tree that included cyanobacteria and alphaproteobacteria, and then replaced the alpha clade with four alternative mitochondria-alphaproteobacteria topologies (TP1-TP4) reported in prior work^59^. The final pruned matrix contained 181 taxa (131 SufE genomes, 24 cyanobacteria/melainabacteria, and 26 mitochondria/alphaproteobacteria). The mito24 alignment contributed 6,749 concatenated amino-acid positions after trimming.

Eight calibrations spanning the root and key crown groups were used: origin of life, origin of oxygenic Cyanobacteria, crown Nostocales, crown Pleurocapsales, red algal Bangiophyceae and Florideophyceae, crown bryophytes, and crown eudicots. Root bounds explored broad windows reflecting early habitability constraints (upper bound 4.5 Ga; lower bounds 4.0, 3.5, or 3.0 Ga, sensu impact chronology and earliest microfossils). Cyanobacterial bounds bracketed literature estimates from geochemical proxies and biomarkers (upper 3.0 Ga^61^; lower 2.32 or 2.50 Ga)^62^. Fossil minima for Nostocales (1.6 Ga)^63^ and Pleurocapsales (1.7 Ga)^64^ followed akinetes and microfossil occurrences. Eukaryotic calibrations followed recent syntheses for red algae, bryophytes and eudicots^59^. The root and cyanobacterial options with two eukaryote schemes (Euk1/Euk2) were combined to generate 12 calibration sets. These, along with four topologies for mitochondria (TP1-TP4) and two clock models (AR/IR; see below), produced 96 dating analyses (Supplementary Information Table S1).

Bayesian relaxed-clock dating was performed in PAML MCMCTree 4.10.7^65^ using both auto-correlated rates (AR) and independent rates (IR) relaxed-clock models. Before formal divergence time estimation, the gradient and Hessian matrices were obtained by using PAML CODEML to improve the accuracy of molecular clock results^66^. Each run involved a burn-in of 2,000 iterations, sampling every 20 steps (20,000 posterior samples). Each configuration was run in duplicate with different random seeds. Convergence and performance were assessed by (i) mcmc3r diagnostics, (ii) infinite-sites test, and (iii) stepping-stone marginal likelihoods to compare topologies, calibrations, and clock models^67^.

#### Ancestral protein reconstruction

IQ-TREE (version 1.6) with the ‘-asr’ option was used to infer the amino acid sequences of ancestral SufE. The best-fit substitution model (LG+C20+I+G4) was chosen based on Akaike information criterion and Bayesian information criterion. The ancestral sequence reconstruction output provided the posterior probability for each amino acid at every site. Sites with a single amino acid having high posterior probability (> 0.30) were directly assigned that residue. For ambiguous sites with lower confidence, top three candidate residues were considered. If the top three residues exhibited similar properties, the highest-probability residue was chosen for that site. If candidates differed in property, the residue predicted to enhance protein solubility and allow the isoelectric point of the protein closer to that of *E. coli* SufE was selected. In cases where a gap (“-”) was the top inference for a site, top four candidates were considered. If none of the four candidate residues yielded a clear improvement in protein property or synthesis, the gap was retained^68^. ASR based on maximum likelihood (ML) method was also inferred by using PAML^65^ and FastML^69^. The amino acid substitution model used in ML was LG.

Representative reference SufS sequences were chosen from diverse taxa, including an extremophilic archaeon (D4gyV5, *Haloferax volcanii*), a Gram-positive bacterium whose SufS interacts with SufU (O32164, *Bacillus subtilis*), and *E. coli* K12, which encodes the canonical SufS (1C0N). SufS sequences were aligned as described above for SufE. A maximum likelihood tree of SufS genes was built from the 131 genomes used for SufE dating, using IQ-TREE, and ASR for SufS was performed in the same fashion as for SufE. Internal nodes corresponding to SufE^LCA^ (2.67 Ga) and SufE^GOE^ (2.17 Ga) were identified on the SufS tree, and the ancestral SufS sequences for these time points were inferred (designated as SufS^LCA^ and SufS^GOE^, respectively). Ambiguous sites in the SufS reconstructions were resolved following the same criteria as those for SufE.

#### AlphaFold3 protein structure prediction

Reconstructed SufS and SufE variant structures were predicted by AlphaFold3^70^ with the ratio of SufS to SufE equal to 2:1. The structure was visualized by ChimeraX. The PLP cofactor of SufS was matched by PDB 5XT6 SufS.

### In vitro experiments

#### Protein preparation

Genes encoding ancestral SufS/SufE proteins (LCA and GOE) were codon-optimized for *E. coli* and synthesized. The present-day *sufS* and *sufE* were PCR-amplified from the genomic DNA of *E. coli* MG1655. The resulting DNA fragments were cloned into pET-30a(+) via *NdeI*/*XhoI* to append a C-terminal His_6_ tag. Since His_6_-SufS^LCA^ construct was insoluble, SufS^LCA^ was prepared by cloning the gene fragment into pMAL-c6T to generate an N-terminal MBP-TEV-SufS^LCA^ fusion. All constructs were sequence-verified (primer lists and sequences in Supplementary Table S3).

Plasmid constructs were transformed into *E. coli* BL21(DE3) pLysS. The strains were grown in LB at 37 °C to OD_600_ ≈ 0.4, and protein synthesis was induced with the addition of 0.4 mM IPTG and subsequent incubation for 5 h at 37°C for SufE^LCA^, SufE^GOE^, SufE^modern^, SufS^GOE^, and SufS^modern^, and for overnight at 18 °C for MBP-SufS^LCA^.

All of the following steps were performed in an anaerobic glove box (O_2_ < 0.1%, Coy) at 4 °C. Cells were harvested, cell pellets were lysed in a buffer solution of 25 mM Tris-HCl, pH 7.6, 150 mM NaCl, 1 mM EDTA, 5 mM DTT and 1 mM phenylmethylsulfonyl fluoride (PMSF), and the lysates were removed (20,000 g, 30 min). His-tagged proteins were purified successively on a HisTrap column (Cytiva) with a 10–500 mM imidazole linear gradient, a HiTrap Q column (Cytiva) using a 50 mM-1 M NaCl linear gradient at a pH of ~2 units above the PI of the protein, and a Superdex 75 Increase 10/300 column (Cytiva) with an elution buffer of 25 mM Tris-HCl, pH 7.6, 150 mM NaCl, 1 mM EDTA, and 1 mM DTT. The MBP fusion protein was purified on an MBPTrap HP column (Cytiva) with maltose elution. The MBP tag was removed by overnight Tobacco Etch Virus (TEV) protease digestion at 4 °C, followed by subtractive Ni^2+^-affinity purification. Protein concentrations were determined by BCA assay against a BSA standard. Aliquots were flash-frozen and stored at −80 °C.

#### Western blotting

Proteins were subjected to SDS-PAGE and transferred to a polyvinylidene fluoride (PVDF) membrane for Western blotting, as previously described^71^, with an anti-His tag mouse monoclonal antibody (ThermoFisher Invitrogen) or anti-MBP monoclonal antibody (LabLead). An HRP-conjugated goat anti-mouse IgG (H+L) secondary antibody (ThermoFisher Invitrogen) was then used, and signals were detected using enhanced chemiluminescence (Thermo Scientific SuperSignal West Pico PLUS) and imaged (Tanon 1600).

#### Cysteine desulfurase activity assay

The cysteine desulfurase activity of the SufS/SufE system was measured using a methylene blue assay as described previously^72,73^. To determine the appropriate SufE concentration, the reaction mixture contained 0.5 μM SufS, a gradient of SufE (0, 0.5, 1.0, 2.0, 4.0, 5.0, 6.0, 8.0, 10.0 μM), and 0.5 mM L-cysteine in 25 mM Tris-HCl buffer (pH 7.4) with 150 mM NaCl. Using GraphPad Prism (v10.4) fit to the Michaelis-Menten equation to obtain the apparent K_m_ of SufE. The formal reaction was conducted at a specified O_2_ level in a modular atmosphere chamber, reaction conditions are 25 mM Tris-HCl (pH 7.4), 150 mM NaCl, 0.5 μM SufS, 2×K_m_ concentrations of SufE, and L-cysteine 0–500 μM. SufS and SufE were pre-equilibrated separately in the chamber at the tested O_2_ level for 30 min and then mixed for 15 min prior to the reaction. The reaction was initiated by adding L-cysteine. After incubation for 30 min at 27°C, the reaction was quenched by adding 0.1 M NaOH. Next, DTT was added to 1 mM final concentration. Then 12.5 μL of 10% zinc acetate was added. Color development reagents, 25 μL of 20 mM DMPD and 25 μL of 30 mM FeCl_3_, were then introduced. The mixture was incubated for 30 min at room temperature in the dark and then shortly centrifuged. The absorbance of the supernatant at 670 nm was measured. One unit (1 mU) of cysteine desulfurase activity is defined as the formation of 1 μmol of S^2−^ per min under the assay conditions.

### In vivo experiments

#### Strain construction

To construct an *E. coli* strain for in vivo evaluation of the role of the reconstructed SufS/SufE proteins on oxidative stress response, we replaced a part of the *isc* operon (*iscUA-hscBA*) with a sequence encoding a ~4.2 kb heterologous mevalonate pathway^74^ in *E. coli* strain MG1655 by using a high-efficiency, low-escape CRISPR/Cas9 genome editing method^75^ with the designed guide sequences (Supplementary Information Table S3). The sgRNA protospacer was introduced by annealing oligonucleotides carrying ~20-bp vector overlaps and assembled into the linearized pTargetT backbone (SpeI-HF). A donor cassette (MVA) flanked by homology arms to the target locus (Δ*iscU*-*hscA*) was assembled by overlap-extension PCR from genomic templates, followed by amplification with primers adding ~20-bp overlaps to the linearized pTargetT vector at 5’ end (Supplementary Information Table S3). pTargetT was linearized by restriction digestion and dephosphorylated (rSAP, NEB). The donor amplicon was inserted by Gibson assembly. All constructs were sequence-verified.

*E. coli* MG1655 cells carrying pCas (Cas9/λ-Red)^76^were prepared at 30 °C. λ-Red was induced with 0.2% L-arabinose, and pTargetT-donor-sgRNA was then introduced into the cells by electroporation. Transformants were selected on LB agar with kanamycin (50 μg mL^−1^) and streptomycin (50 μg mL^−1^) in the presence of L-arabinose and/or MVA as indicated. Colonies were screened by PCR (Supplementary Information Table S3), and positive clones were confirmed by Sanger sequencing. pTargetT was cured by repeated passage in medium without streptomycin. pCas was subsequently cured by growth at 42 °C in the absence of antibiotics. The resulting strain was denoted *E. coli* MG1655 Δ*iscU*-*hscA::MVA*^*+*^ (CHY97).

To ensure that CHY97 closely matched the genetic background of other strains used for phenotype testing (WO539/WO541 from the W. Outten lab, see below), P1 phage transduction was used to move the MVA cassette from WO539/541 into the strain. In the WO539/541 strains, the *PBAD-MVA-kan*^*R*^ cassette was integrated at the *lac* operon locus under the control of a BAD promoter and included a kanamycin resistance marker for selection^26^. P1 phage prepared on donor strain WO541 was transduced into CHY97, selecting for *kan*^R^ colonies. This yielded strain CHY98 (genotype MG1655 Δ*iscUAhscBA::MVA*^*+*^ *+ lac::[MVA+, kan*^*R*^*]*), which carries the arabinose-inducible MVA pathway integrated at the *lac* locus, identical to WO539/541. CHY98 was used as a positive control strain in growth assays, as it retains the native *suf* operon and has the MVA pathway just like the WO539/541 mutants, allowing us to distinguish effects of the complementation constructs from any unintended effects of the MVA system or antibiotic markers.

The donor and base strains used in this study: *E. coli* WO539 (MG1655 Δ*iscU-fdx* Δ*sufSE::cm*^*R*^ *MVA*^*+*^*-kan*^*R*^) and WO541 (MG1655 Δ*iscU-fdx* Δ*sufE::cm*^*R*^ *MVA*^*+*^*-kan*^*R*^) were constructed by multiple P1 transductions of mutations from previously constructed strains. Briefly, *iscUA-hscBA-fdx* was replaced with *kan*^R^ from pKD4 and then the *kan*^R^ was removed with plasmid pCP20 expressing flippase (FLP) recombinase^77^. The *MVA*^*+*^-*kan*^R^ locus was then P1 transduced into the Δ*iscU*-*fdx* background. Finally, the *sufE* gene or *sufSE* genes were replaced with *cm*^R^ from pKD3 and then P1 transduced into the Δ*iscU-fdx MVA*^*+*^-*kan*^R^ background. The final transductants were selected on and maintained in “5-component SB medium” (see below) to ensure that they had the mevalonate supplement for initial recovery. This Super Broth (SB) was a rich medium (32 g L^−1^ tryptone, 20 g L^−1^ yeast extract and 5 g L^−1^ NaCl), supplemented with 0.2% (w/v) L-arabinose, 0.4% (w/v) D-glucose, 50 mg L^−1^ chloramphenicol, 50 mg L^−1^ kanamycin and 300 μM mevalonolactone (MVA, CAS 674-26-0).

#### Complementation assays

To complement the SufE/SufSE deletions in vivo, a series of arabinose-inducible plasmids carrying various combinations of present-day or ancestral *sufE* and *sufS* genes were constructed. For the single-gene constructs (pBAD/His B-SufE series), the coding sequences for SufE^LCA^, SufE^GOE^, and SufE^modern^ were PCR-amplified (Supplementary Information Table S3) from their respective pET 30a(+) templates and cloned into pBAD/His B. This placed the *sufE* gene under the arabinose promoter, with an N-terminal His_6_-tag (from the vector) on the expressed SufE protein. For the *sufS/sufE* constructs (pBAD/His B-SufSE series), two genes were amplified in tandem, including their native intergenic spacer. In the case of the *sufS*^*modern*^ and *sufE*^*modern*^, they were amplified directly from *E. coli* MG1655 genomic DNA using primer pair pBAD-SufSE-F and HindIII-His-SufE-R (Supplementary Information Table S3). For the *sufS*^*GOE*^*/sufE*^*GOE*^ and *sufS*^*LCA*^*/sufE*^*LCA*^ constructs, the *sufS* and *sufE* fragments were assembled by overlap extension PCR to include the native intergenic spacer (Supplementary Information Table S3). The two PCR fragments (ancestral *sufS* and s*ufE*) were then joined by overlap PCR to create a full-length *sufSE* di-cistron fragment with spacer and tags. This fragment was digested and cloned into pBAD/His B. Similarly, for SufS^LCA^/SufE^LCA^ adding MBP at N terminal, a bicistronic insert was generated where *sufS*^*LCA*^ included the coding sequence for an MBP tag fused at its N-terminus (carried over from the pMAL-SufS^LCA^ template, Supplementary Information Table S3). Thus, this construct tests the effect of co-expressing an MBP-tagged SufS^LCA^ with SufE^LCA^ in vivo. All pBAD constructs were verified by restriction mapping and sequencing. The resulting complementation plasmids were each introduced into the appropriate mutant background. Successful transformants were selected on 5-component SB with 50 mg L^−1^ ampicillin (to maintain the pBAD plasmid, referred to as “6-component SB), and verified. Glycerol stocks were made for each complementation strain.

#### Growth assays under oxidative stress

The growth phenotypes of the complemented strains under various oxidative stress conditions were assessed. The phenazine methosulfate (PMS) could generate oxidative stress inside cells. Concentration ranges for these stressors were determined based on literature and our pilot experiments: PMS was used at 0, 30, 60, 90, 150, 240, 300, and 450 μM^27^.

For each strain, growth curves were obtained using a 96-well microplate format (200 μL culture per well, in at least three replicates). Prior to the experiment, all strains were revived from glycerol stocks on LB agar and then pre-cultured in a “6-component SB medium” (see above) to ensure they had the mevalonate supplement for initial recovery. After overnight growth in 6 component SB (37 °C, shaking), cultures were diluted 0.5% into the same medium but without mevalonate in order to test if the pBAD plasmids restored Fe-S dependent (MVA independent) isoprenoid biosynthesis. This medium still had ampicillin. We chose a 0.5% inoculum to minimize carryover of MVA from the starter culture. At this low inoculation, the control strain with empty plasmid (which cannot synthesize isoprenoids without MVA) did not grow for at least 48 h, confirming effective depletion of intracellular MVA with no carryover from the starter culture.

Growth assays were performed in a FLUOstar Omega microplate reader (BMG Labtech) at 37 °C with continuous orbital shaking (200 rpm). The optical density was recorded every 5 min. Due to PMS’s color change upon oxidation (the medium gradually turned green, and even cell growth itself can alter PMS’s absorbance), a detailed spectral analysis (scanning 450–800 nm) showed that PMS causes elevated absorbance across a broad range (480–750 nm) and that high cell density plus PMS can increase OD readings in a wavelength-dependent manner. However, above 750 nm, the OD signal from cells alone started to diminish (due to scattering properties) and at wavelengths below 480 nm, the artifact due to PMS oxidation was minimal. OD_450_ nm for PMS experiments was applied, as 450 nm gave a reliable indication of cell growth while avoiding most interference from PMS oxidation^78^. In all experiments, the first few OD readings (prior to significant growth, *e.g*. the mean of 0–5 min readings) were used as a blank and subtracted from subsequent readings for each well, to normalize starting OD.

Each growth curve was then analyzed to extract key parameters. The R package gcplyr (v1.X) was utilized for growth curve analysis^79^. The OD data (log-transformed OD values vs. time) were fit to a growth model to estimate: the lag time (adjustment phase duration), the maximum growth rate (slope during exponential phase), and the maximum cell density (OD). The results were plotted using GraphPad Prism.

#### Parallel Reaction Monitoring (PRM) Targeted proteomics

Synthesis of SufS and SufE in complementation cells was confirmed by targeted quantitative proteomics using PRM mass spectrometry. Cultures of the WO539 + pBAD/His B-SufSE (ancestral sufSE) strains were grown to log phase (OD_600_ ~0.5) and stationary phase (overnight culture) in 6 component SB medium (with Amp and MVA) under aerobic conditions. Cells from 50 mL of each culture were harvested by centrifugation (5,000 g, 10 min, 4 °C), washed with cold PBS, resuspended in 200 μL of urea lysis buffer (7 M urea, 2 M thiourea, 0.1% CHAPS, 1× protease inhibitor cocktail), and lysed by sonication on ice (15 min total sonication time, in pulses). Total protein concentration in each sample was determined by BCA assay using a standard protocol^80^. An aliquot (~50 μg protein) of each sample was subjected to digestion and subsequent mass spectrum (MS) by following a filter-aided sample preparation (FASP) protocol^81^.

For mass spectrometry, dried peptides (~0.5 μg) were analyzed by LC-MS/MS on an EASY-nLC 1200 UHPLC system (Thermo Fisher Scientific) coupled to an Orbitrap Exploris 240 mass spectrometer (Thermo Fisher Scientific). Separation was achieved on a C18 reversed-phase column (150 μm × 150 mm, 1.9 μm) using a 60 min gradient (8–45% acetonitrile/0.1% formic acid) at 500 nL min^−1^. The mass spectrometer was operated in positive ion mode with full MS scans acquired at 120,000 resolution (m/z 350–1500), followed by higher-energy collisional dissociation (HCD) with normalized collision energy 30%. MS2 spectra were acquired in the Orbitrap at 15,000 mass resolution, with dynamic exclusion (45 s). The DDA MS/MS data were searched using Proteome Discoverer (v2.3, Thermo) against the UniProt *E. coli* K-12 proteome reference (downloaded 2024-06-11) with the sequences of SufS^GOE^/SufE^GOE^ and SufS^LCA^/SufE^LCA^ proteins appended (total 4406 sequences). Trypsin (up to 2 missed cleavages allowed) and Carbamidomethyl (Cys) as fixed, and Oxidation (Met) and Acetyl (protein N-terminus) as variable modifications were employed. Precursor ion mass tolerance was 10 ppm, and fragment ion tolerance 0.02 Da. Peptide-spectrum matches were filtered to a false discovery rate (FDR) of < 1% using a target-decoy strategy.

Using Skyline (MacCoss Lab, v4.X), 3 unique peptides for each target protein (SufS variants and SufE variants of interest) that were confidently identified in the DDA run were selected. The precursor m/z for each target peptide and its charge state, along with the retention time (RT) window (~5 min around the observed RT), were compiled for PRM. In total, 12 peptides covering all target proteins were monitored by unique peptides. PRM assays were performed by measuring these peptides and corresponding stable isotope-labeled standards (SIS). Quantification was achieved by calculating the ratio of protein peptides to SIS peptide peak areas.

## Supplementary Material

Supplementary Files

This is a list of supplementary files associated with this preprint. Click to download.

- SIGuide.docx
- SupplementaryInformationTable1.xlsx
- SupplementaryInformationTable2.xlsx
- SupplementaryInformationTable3.xlsx
- ExtendedDataFigures.docx

Supplementary information is available for this paper.

## Data Availability

All data generated or analysed during this study are included in this published article, its Supplementary Information files and https://github.com/eacochen/SufE.
